# Serrated Polyposis: An Enigmatic Model of Colorectal Cancer Predisposition

**DOI:** 10.4061/2011/157073

**Published:** 2011-05-30

**Authors:** Christophe Rosty, Susan Parry, Joanne P. Young

**Affiliations:** ^1^Pathology Queensland and UQ Centre for Clinical Research, Royal Brisbane and Women's Hospital, Herston, QLD 4029, Australia; ^2^Familial Cancer Laboratory, QIMR, 300 Herston Road, Herston, QLD 4006, Australia; ^3^New Zealand Familial Gastrointestinal Cancer Registry, Auckland City Hospital, Auckland 1142, New Zealand; ^4^Department of Gastroenterology and Hepatology, Middlemore Hospital, Auckland 1640, New Zealand; ^5^The University of Queensland School of Medicine, Herston, QLD 4006, Australia

## Abstract

Serrated polyposis has only recently been accepted as a condition which carries an increased personal and familial risk of colorectal cancer. Described over four decades ago, it remains one of the most underrecognized and poorly understood of all the intestinal polyposes. With a variety of phenotypic presentations, it is likely that serrated polyposis represents a group of diseases rather than a single entity. Further, neoplastic progression in serrated polyposis may be associated with premature aging in the normal mucosa, typified by widespread gene promoter hypermethylation. From this epigenetically altered field, arise diverse polyps and cancers which show a range of molecular features. Despite a high serrated polyp count, only one-third of colorectal cancers demonstrate a *BRAF* V600E mutation, the molecular hallmark of the canonical serrated pathway, suggesting that though multiple serrated polyps act as a marker of an abnormal mucosa, the majority of CRC in these patients arise within lesions other than BRAF-mutated serrated polyps.

## 1. Introduction

**Figure 1:**
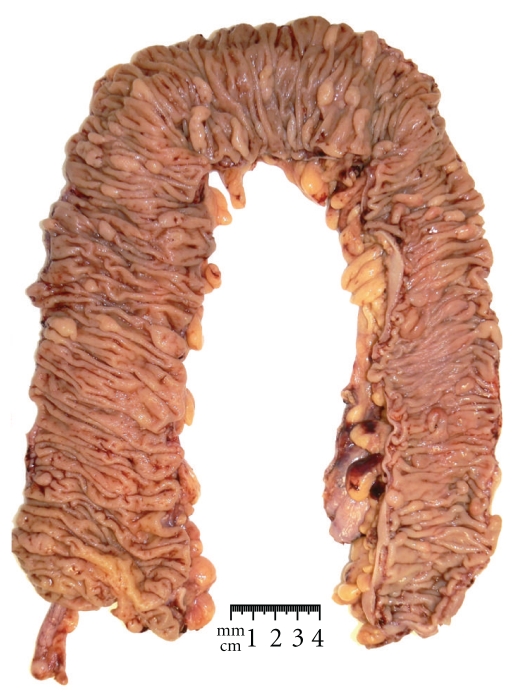
Colectomy specimen from a patient with serrated polyposis showing multiple flat polyps on mucosal folds, measuring less than 10 mm, distributed throughout the colon (courtesy of Dr. Andrew Clouston, Envoi Pathologists, Brisbane).

Serrated polyposis [1], a condition also known as hyperplastic polyposis, was described in the early seventies [2] and remains to the present day the most under-recognized and least understood of the colorectal polyposes. Its defining characteristic, specifically the presence of numerous colorectal epithelial polyps with serrated architecture (Figure 1, Text Box 1), placed it, until relatively recently, among conditions without significant clinical consequence, based upon the perception that all serrated polyps were innocuous. For decades, the malignant transformation of conventional adenomas was considered to be the single mechanism underlying the genesis of colorectal cancer (CRC) [3]. In the late nineties, a number of important observations set in motion a major paradigm shift in the way the initiation and progression of CRC were viewed. These observations carefully detailed at a molecular level that some serrated polyps may act as the precursor lesions in an alternative developmental pathway for CRC, existing alongside the traditional adenoma-carcinoma sequence [4]. As with the histological observations suggesting that a subset of serrated polyps may develop features associated with malignancy [5–7], clear molecular evidence for the malignant transformation of serrated polyps was also first observed in a patient with serrated polyposis [8], suggesting that this condition could serve as a model for the malignant conversion of serrated polyps, a mechanism which has become known as the *serrated pathway*. Today the clinical significance of serrated polyposis rests upon consistent observations in relatively large studies of an increased personal and familial risk of CRC [9–12]. To include serrated polyposis as a CRC predisposition is a concept whose time has come [13].

**Serrated polyps or hyperplastic polyps? [1].**
Epithelial polyps with serrated architecture arising in the large intestine were until very recently collectively known as
hyperplastic polyps. A modern classification has now been proposed which uses the descriptive umbrella term serrated
polyps for all epithelial polyps with serrated architecture and the term hyperplastic polyps for the subset of small
common lesions without evidence of abnormal proliferation. A detailed description of the WHO classification
of serrated polyps is given in Section 3 of this paper.

## 2. Definition and Features

Clinical criteria for the recognition of serrated polyposis were first established in 2000 [14] and were proposed for two major reasons. Firstly, to delineate it from the clearly penetrant and clinically severe condition familial adenomatous polyposis (FAP). Secondly, necessarily stringent criteria ensured that diminutive serrated polyps observed relatively often in the distal colorectum of older patients, including those which cluster around rectal cancers, were not included in the definition [15, 16]. In serrated polyposis, the serrated polyps demonstrate features that distinguish them from sporadic serrated polyps in that they are unusually numerous. They also may be large and proximal and may exhibit atypical histological architecture. While large polyps are preferentially located in the proximal colon, small sessile polyps are often distributed throughout the colorectum [14, 17]. An example of the gross appearance of a serrated polyposis colon is shown in Figure 1.

The current revised criteria, published in 2010 [1], are

1. at least five serrated polyps proximal to the sigmoid colon with two or more of these being >10 mm,
2. any number of serrated polyps proximal to the sigmoid colon in an individual who has a first-degree relative with serrated polyposis,
3. >20 serrated polyps of any size, but distributed throughout the colon. The implied meaning of this last criterion is that the polyps are not all present in the rectum (Text Box 2).

The individual criteria to define serrated polyposis have been difficult to delineate due to the phenotypic plasticity of this condition, which overlaps with the occurrence of common sporadic serrated polyps found in the population. Serrated polyposis may represent a group of diseases or a continuum which is influenced by a variety of genetic and environmental modifiers, rather than a single disease. Therefore, at the time of writing, the current criteria remain empirical in nature [1].

**Clinical criteria for the identification of serrated polyposis [1].**
(1) At least five serrated polyps proximal to the sigmoid colon with two or more of these being >10 mm.
(2) Any number of serrated polyps proximal to the sigmoid colon in an individual who has a first-degree relative
   with serrated polyposis.
(3) >20 serrated polyps of any size, but distributed throughout the colon.

When establishing the serrated polyp count for an individual, Higuchi and Jass have suggested that the polyp count be cumulative over time [18]. Serrated polyposis affects both sexes and is most likely to be identified in persons aged between 55 and 65 years. It may become apparent considerably earlier [7, 19–21], particularly if symptoms such as bleeding, bowel habit alterations, and abdominal pain result from the polyp burden or advanced neoplasia. The range of ages at presentation in the literature varies from 11 to 83 years [7, 19–21]. 

Estimates of the prevalence of serrated polyposis suggest that it is relatively rare. A large population-based screening trial of over 40,000 asymptomatic patients aged 55–64 years prospectively identified serrated polyposis at a rate of 1 in 3000, with 50% of these demonstrating at least one synchronous conventional adenoma [22]. Rubio et al. reported that only 10 cases were observed in a 1026-bed Scandinavian hospital over a 16-year period [23], whilst Leggett et al. identified 12 cases from a similar institution during a 5-year period [24]. A family history of CRC is a relatively frequent finding with figures of up to 59% reported [25–27], though other publications suggest that this is rare [28, 29]. A recent large multicentre study from The Netherlands has estimated the risk to first-degree relatives of CRC to be approximately fivefold that of the general population adjusted for demographic variables: RR 5.4 (95% CI 3.7 to 7.8) [10]. Studies of ethnicity suggest that, in contrast to conditions such as FAP and Lynch syndrome which can occur in many ethnic groups, serrated polyposis is a condition largely of north-western Europeans. Observations from a multiethnic New Zealand gastroenterology service demonstrated that, in a 24-case series of serrated polyposis patients, all cases were derived from the European component, despite only 46% of the attending patients having European ancestry [17]. Buchanan et al. reported a prevalence of 95% northern Europeans in a case series of 126 serrated polyposis patients [11]. Similarly, Kalady et al. reported that, in a large series of serrated polyposis patients (*n* = 115) collected in Ohio, 97% were white [27]. The fundamental defect in serrated polyposis has yet to be elucidated and may involve defects in inflammation and/or apoptosis. The involvement of widespread DNA methylation in the normal mucosa of patients with this condition [30] suggests deregulation of an epigenetic control mechanism, either directly or as a consequence of upstream genetic events.

## 3. History of Its Recognition as a Colorectal Cancer Predisposition

Described in some earlier reports primarily in order to distinguish it from FAP, serrated polyposis was originally considered to have no important clinical consequences [29]. However, today it is recognized as a condition with substantial risk of CRC. The early literature contains much to interest those who study serrated polyposis, and the serrated pathway to carcinoma in general. Serrated polyposis was described as early as 1970 by Goldman et al. [2]. In this case report, a 42-year-old man presented with 30 serrated polyps ranging in size between 0.75 and 1.5 cm. Of particular interest was the observation of adenomatous transformation within the polyps, a concept now readily accepted, but somewhat controversial in those earlier times. Despite several reports relating serrated polyps to the development of adenomatous change, villous components, and even adenocarcinoma [31–33], the bulk of investigations published around this time returned a classification of nonneoplastic for serrated polyps [3, 34–36]. 

 In 1978, Cooke described serrated polyposis as a variant of FAP [5]. A further report describing cancer and dysplasia in a background of serrated polyposis was presented in 1979 [37]. In this publication, the authors, whilst suggesting that hyperplastic polyps were “benign, nonneoplastic proliferations which unlike tubular and villous adenomas did not predispose the patient to colonic cancer,” went on to demonstrate a case of serrated polyposis where hyperplastic adenomatous transformation and cancer “had probably occurred,” and recognized that cancer could be associated with unusual cases of multiple hyperplastic polyps [6]. In 1980, seven cases of serrated polyposis were recorded from a London hospital [29], and this became an influential landmark paper in the confusion surrounding serrated polyposis. Six of the seven cases were male, and there was an average age at presentation of 37 years. The presence of larger metaplastic polyps was noted, and the possibility that “metaplastic polyposis” was a pathological entity was raised [29]. With followup, however, no cases of CRC were observed, thereby designating serrated polyposis as a low-risk condition of young males. This paper influenced thinking on serrated polyposis for over a decade. However, during the 1990s association with CRC was revisited by Torlakovic and Snover [7], Burt and Samowitz [38], and Jeevaratnam and colleagues in a familial setting [39] and in a small series of patients who were instrumental in demonstrating the malignant transformation of serrated polyps [8]. Many series and case reports have now been published [5–8, 19–21, 23, 24, 26, 28, 39–60]. In the three largest series published to date, 25–38% of patients presented with at least one CRC [9, 12, 27], and multiple CRCs were common [11]. However, the problem remains that published series are predominantly composed of retrospective clinic-based records, and therefore the estimation of CRC risk is likely to be inflated and to reflect a CRC risk associated with symptomatic patients. In addition, the wide variety of phenotypic presentations within serrated polyposis has the potential to be associated with varying risk magnitudes. At the time of writing, no prospectively collected population-based risk estimates for CRC are available. A summary of the findings in published series of serrated polyposis cases is presented in Table 1.

## 4. Serrated Polyp Subtypes

Serrated polyps are the second most common type of colorectal polyps, after conventional adenomas, found during population colonoscopy. Serrated polyps are also the most prominent phenotypic feature in serrated polyposis usually ranging in number from 5 to greater than 150, and varying greatly in size. In addition, a diverse range of dysplastic lesions of both conventional and serrated lineages may also be present. 

In a study reporting the prevalence of each polyp type diagnosed from 179,111 consecutive population colonoscopies in the United States, Lash and colleagues found that epithelial benign polyps were classified as conventional adenomas in 58.6% and as serrated polyps in 41.4% [62]. The terminology and histologic classification of serrated polyps have been a matter of debate for some years. The most clinically relevant feature is the presence of dysplasia that increases the risk of developing CRC and impacts colonoscopy surveillance intervals. Therefore, the classification of serrated polyps into dysplastic polyps and nondysplastic polyps is the most meaningful division. However, there is still some degree of confusion in diagnosing serrated polyps with reported significant variation in the detection rate and in the histologic classification, justifying the need for increased awareness and education [63]. All of the following subtypes are observed in serrated polyposis.

### 4.1. Nondysplastic Serrated Polyps

Nondysplastic serrated polyps comprise hyperplastic polyps and sessile serrated adenomas/polyps, representing the vast majority of all serrated polyps. The use of new high-definition endoscopes in association with chromoendoscopy or narrow-band imaging has led to a higher detection of these polyps.

#### 4.1.1. Hyperplastic Polyps (HPs)

More than 75% of serrated polyps are HPs [62, 64, 65]. Most often measuring 5 mm or less, HPs are sessile pale lesions, usually found on the tip of mucosal folds in the distal colorectum, with normal architecture and normal proliferation characteristics. In the proximal colon, HPs are often larger and more difficult to visualize. The prevalence of HPs in asymptomatic adults aged 40 years or more has been estimated to be around 10% in Western populations [66–68]. In autopsy studies, the prevalence rate of HPs ranged from 5% in a Cretan study to 40% in a British study [69–71]. While HPs develop at an earlier age than conventional adenomas, its incidence does not seem to significantly increase after 50 years, contrasting with the positive correlation between increased age and the prevalence of conventional adenoma [72].

Histologically, HP is the prototypical example of serrated polyps of the colon with a saw-toothed appearance caused by in-foldings of the crypt lining epithelium in the upper half of the crypts. All types of HP are characterized by elongated crypts, with maturation of cells towards the surface, and proliferation activity limited to the lower portion of the crypts. HPs are further divided into 3 histologic subtypes: microvesicular, goblet-cell, and mucin-poor, without clinical relevance as yet. Microvesicular HP is the most common type, characterized by the presence of columnar cells with abundant apical vesicular mucin and by a decreased number of goblet cells. In contrast, goblet-cell HPs show elongated crypts with numerous goblet cells and minimal serration limited to the most upper portion of the crypts. These polyps may be overlooked and are often underdiagnosed and therefore may occur more frequently than reports suggest. Finally, mucin-poor HPs are very rare and display prominent serration, regenerative changes, and mucin-depleted columnar cells. It is still debated whether mucin-poor HPs are a true separated subtype or an irritated form of microvesicular HPs.

#### 4.1.2. Sessile Serrated Adenomas/Polyps (SSA/P)

First designated “serrated polyps with abnormal proliferation” by Torlakovic et al. in 2003, SSA/Ps comprise 15–20% of all serrated polyps [65]. SSA/Ps are flat or slightly elevated lesions most commonly found in the proximal colon and usually measuring more than 5 mm. Histologically, SSA/Ps differ from HPs by the presence of abnormal architectural features secondary to abnormal proliferation. Whereas the proliferative zone in HPs is located in the base of the crypts, it is usually on the sides of the crypts in SSA/Ps, leading to asymmetrical growth in crypts with an inverted T shape or L shape. Other histologic features of SSA/Ps include the presence of mature goblet cells at the bases of the crypts, hyperserration at the base or throughout the crypts, and pseudoinvasion of the muscularis propria. Dysplasia is not present.

### 4.2. Dysplastic Serrated Polyps

#### 4.2.1. Traditional Serrated Adenomas (TSAs)

TSA is a relatively uncommon polyp, comprising up to 5% of serrated polyps in Western countries with a higher prevalence in Asia, particularly in Korea [73]. Compared to SSA/Ps, TSAs are protuberant polyps more frequently found in the left colon and in older individuals. The architecture of TSA is often more complex than villous or tubulovillous adenoma, with prominent serration and finger-like projections. The presence of ectopic crypt foci in TSAs, defined by the crypts with their base not seated at the level of the muscularis propria, is useful to distinguish them from SSA/P [74]. The neoplastic cells are characterized by an abundant eosinophilic cytoplasm and elongated pencillate nuclei. Dysplasia in TSA is usually mild, with a different appearance of the dysplasia associated with conventional adenoma and low proliferative characteristics. However, conventional adenoma-type dysplasia can also be present and sometimes with high-grade features.

#### 4.2.2. Sessile Serrated Adenomas/Polyps with Dysplasia (SSA/P-D)

**Figure 2:**
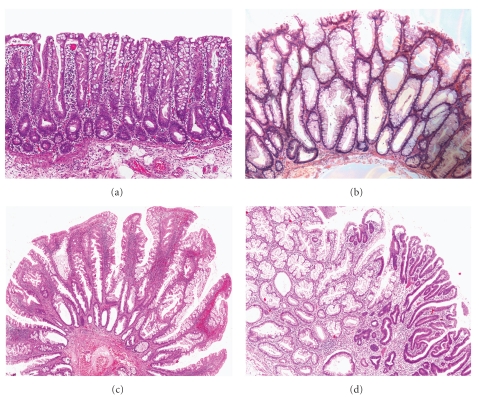
Showing 4 serrated polyp subtypes. (a) Microvesicular hyperplastic polyp with crypt serration and proliferative crypt bases. (b) Sessile serrated adenoma/polyp, showing asymmetrical serrated crypts and dilated crypts. (c) Traditional serrated adenoma, with prominent complex serration and hypereosinophilic cells. (d) Sessile serrated adenoma/polyp with high-grade dysplasia (right).

The presence of dysplastic crypts in a SSA/P was often reported as part of the “mixed polyp” group and is now better recognized as a specific category of dysplastic serrated polyps with malignant potential. In most cases, dysplasia in SSA/P is similar to dysplasia in conventional adenoma and is well demarcated from the nondysplastic areas. Dysplasia in SSA/Ps is rare, found in 14% of all SSA/P in a recent study by Lash et al. [62]. In this study of over 2000 patients, the median age for presenting with a nondysplastic SSA/P was 61, increasing to 66 for SSA/P with low-grade dysplasia, 72 for SSA/P with high-grade dysplasia, and 76 for SSA/P with invasive carcinoma (a span of 15 years). In contrast, the span between tubular adenoma and non-SSA/P carcinoma is only 5 years. Examples of serrated polyp subtypes are given in Figure 2.

## 5. Phenotypic and Molecular Heterogeneity

Table 2 shows the rates of molecular alterations for the major serrated polyp subtype categories, after adding all available results from various large studies [73, 75–84]. Somatic molecular alterations associated with serrated polyps have been well described in previous publications [54, 76, 79, 85–87] and include *BRAF* (V600E) mutation, *KRAS* (codons 12 and 13) mutations, *MLH1* methylation, *MGMT* methylation, and CpG island methylator phenotype (CIMP). The prevalence of these alterations varies according to the subtype of serrated polyp. *BRAF* mutation is the most common alteration in all polyp types, with the highest rate in SSA/Ps (83.9%) and the lowest rate in goblet-cell HPs (20%). In contrast, *KRAS* mutation is most commonly detected in goblet-cell HPs (48.4%) and TSA (22.4%). *MLH1* methylation ranges from 14.3% in goblet-cell HPs to 47.5% in TSA. Interestingly, while *MLH1* methylation does occur quite frequently, a high-level microsatellite instability phenotype is very rarely encountered in serrated polyps [76, 88]. MSI-H is a late and probably an important pivotal event in the serrated pathway, occurring at the transition between high-grade dysplasia and invasive carcinoma [76, 89]. *MGMT* methylation ranges from 0% in goblet-cell HPs to 74.2% in TSA. A high level of CIMP, defined by ≥2/5 methylated markers, occurs in 39% of HPs, 76% of SSA/Ps, and 79% of TSA [76].

The question of phenotypic heterogeneity in serrated polyposis can be further considered with an examination of the reported molecular changes of serrated polyps as they apply to the syndromic patient. Though there appear to be at least two phenotypic subtypes which correspond to the first and last criteria (Text Box 2), a previous report from the UK clearly demonstrates that the predominant molecular change in the polyps is that of *BRAF* mutation, even in patients with numerous polyps [25], and therefore suggesting that oncogene mutations are of little value in subtyping this disorder. However, rare cases of serrated polyposis are reported where *KRAS* mutations predominate [25], and current evidence suggests that, in at least *some* of these patients, biallelic germline mutation of *MUTYH* may be responsible [25, 26, 90, 91].

Although the possibility of these two types of serrated polyposis was first raised over 10 years ago [92], the application of such a classification to CRC risk may not be readily implemented. Even though large and dysplastic polyps are likely to be an indicator of high malignant potential, the presence of CRC in cases with multiple small HPs [61], as well as the absence of CRC in many cases of serrated polyposis [11, 12] with large and dysplastic polyps, argues against a nonoverlapping classification.

## 6. Epigenetic Field Defect in Establishment of Neoplasia

CRC in general develops through one of two independent molecular pathways that involve sequences of genomic and epigenomic alterations associated with pathological and clinical features: the adenoma pathway in 70–80% and the serrated pathway in the remaining 20–30%. The somatic molecular features which characterize the serrated pathway to CRC include activating mutations in *BRAF *[81, 83, 86] and widespread hypermethylation of gene promoters (CIMP) [87] with or without MSI [8, 42]. In the serrated pathway, the earliest known event is somatic *BRAF* mutation, found in aberrant crypt foci [93], and with a high rate in microvesicular HP and SSA/P [76, 85]. The hypermature cells of the upper crypt in serrated polyps are thought to result from a mechanism of oncogene-induced senescence brought about by the presence of an activating *BRAF* mutation [94]. Escape from senescence is achieved subsequently by the inactivation by promoter methylation of tumour suppressors controlling senescence [95], thus allowing a lesion to progress to a more proliferative neoplasm. An epigenetic field defect present in serrated polyposis would facilitate this process more readily [30] with the consequent increase in polyp numbers which define the condition. When multiple lesions are examined, serrated pathway features of *BRAF* mutation and CIMP demonstrate a high rate of concordance between discrete lesions in individuals with serrated polyposis [86, 87]. As would be expected, most CRCs (70%) in serrated polyposis derive from the proximal colon [17]. An exception to this occurs in young-onset patients (<50 yrs old) where the CRCs are more likely to be distal [11, 12, 26]. This is an under-recognized feature of young-onset serrated polyposis though it has been mentioned in previous publications [96].

 Hypermethylation of gene promoters is also observed in the normal mucosa of individuals with increasing age [97, 98] and is also more likely to be associated with synchronous proximal CRC with concordant molecular features [99, 100]. However, in serrated polyposis, increased methylation of gene promoters is evident even in the normal mucosa of younger individuals [87, 91, 101], indicating that an epigenetic regulatory defect may be present in the normal tissues of these patients and suggesting a prematurely aged mucosa associated with increased risk for the establishment of neoplasia. Of interest, in 1968, Arthur observed that metaplastic polyps were a marker of age in the normal mucosa [35]. The concept of an epigenetic field defect in serrated polyposis was clearly demonstrated by Minoo et al. in 2006 [30]. Significantly the level of methylation in apparently normal mucosa was higher in serrated polyposis patients when compared to patients with sporadic serrated polyps.

## 7. Role of the Conventional Adenoma in Cancer Risk

The complex biology of serrated neoplasia and the plasticity of its developmental pathway can give rise to CRC with variable MSI status [8], and to small numbers of apparently conventional adenomas, in addition to multiple serrated polyps. It has been estimated that conventional adenomas are seen in up to 90% of serrated polyposis patients [23, 24], raising the notion that lesser numbers of conventional adenomas are part of the syndrome. Importantly, the risk for patients with serrated polyposis to present with a synchronous CRC at time of diagnosis is significantly higher when at least one conventional adenoma is present [11, 12, 24].

**Figure 3:**
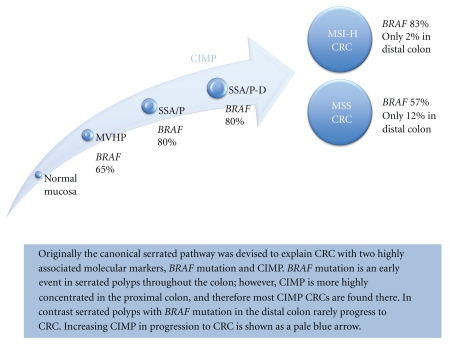
The canonical serrated pathway showing progression through MVHP (microvessicular hyperplastic polyp) to SSA/P (sessile serrated adenoma/sessile serrated polyp) and SSA/P-D (SSA/P with dysplasia) to CRC with CIMP and high levels of BRAF mutation frequently arising in the proximal colon. CRC with CIMP can evolve into MSI-H and non-MSI-H subtypes. Though KRAS mutation can be observed in CIMP CRC, these are relatively rare [103, 104].

Adenomatous lesions in a serrated polyposis patient may either evolve from serrated polyps, progressing to a conventional type of dysplasia, or arise via an alternate mechanism. The presence of “mixed polyps,” as they were previously known (now called SSA/P with dysplasia), which demonstrate a very high rate of somatic *BRAF* mutation (80–90%) [76] and therefore, by implication, origin in a serrated polyp, provides a plausible precursor lesion for the CRC which arises in serrated polyposis [6, 19, 50, 56, 57]. In contrast, conventional adenomas almost never harbor a *BRAF* mutation [84]. If most of the CRCs in serrated polyposis were to arise from advanced serrated polyps, a high rate of *BRAF*-mutated CRC would be expected. CRCs in serrated polyposis have shown somatic *BRAF* mutation in 33% of 6 cases in an early published report [86] and, consistently, in 19/58 (33%) of a recent case series, [102]. Whilst this level is still greater than that of a population series, (which ranges from 7% in southern Europeans to 21% in Anglo-Celts [103]), these observations suggest that the majority of CRCs arising in serrated polyposis develop within lesions not known to be involved in the *canonical serrated pathway* (see Figure 3).

The balance of CRC (BRAF wild-type) in serrated polyposis either demonstrates somatic *KRAS* mutation at a rate of approximately 19% which is half that of the population [102] or is oncogene mutation null. These CRCs may arise, in the manner of common CRC, from the conventional adenomas which frequently coexist in patients with serrated polyposis [17, 23]. Of interest, in reporting the first autosomal dominant family with serrated neoplasia, Jeevaratnam et al. made note of an adenomatous precursor in contiguity with a small CRC [39] and concluded that the CRCs in serrated polyposis arise through both the development of dysplasia in serrated polyps and through coincident conventional adenomas. In 2010, Pai et al. published a report which examined the adenomas which coexisted with SSA/P in the general (nonsyndromic) population [105]. They observed that 35% of the polyps removed from population patients with an index SSA/P were conventional adenomas. Pai et al. observed 3 morphologic features which were more prevalent in the study adenomas compared to control adenomas in a population without SSA/P: eosinophilic cytoplasm, focal (rather than widespread) serration, and crypt dilatation. These features were seen in 30% of the study adenomas compared to 2.5% of controls. In addition, these atypical polyps demonstrated low levels of methylation, and increased staining for MUC6, properties more associated with serrated lesions than with adenomas. Importantly, none of these lesions showed *BRAF* mutation. Given previous findings from the same group of authors which demonstrated that patients with an index SSA/P had a significantly increased risk of having other serrated polyps, combined with the presence of atypical conventional adenomas, supports the presence of an epigenetic field defect in serrated polyp patients including those not meeting the criteria for serrated polyposis [105, 106].

Small numbers of serrated polyps as well as CRCs with somatic *KRA*S mutation are also observed in serrated polyposis patients. Somatic *KRAS* mutation straddles the division between serrated and adenomatous polyps and is mutually excluded in lesions bearing *BRAF* mutation [107]. It is observed in goblet-cell HPs [85] which rarely undergo malignant transformation; however, its presence, albeit less frequently, in advanced serrated polyps [84], in rare contiguous serrated polyps attached to population CRC (Young, unpublished observations) and in the lesions present in biallelic mutation carriers for *MUTYH *[90], suggests that serrated lesions with *KRAS* mutations are not completely devoid of malignant potential.

## 8. The Smoking Paradox and Serrated Polyposis

Risk factors for the development of serrated polyps in the population are similar to risk factors for the development of conventional adenomas, including alcohol consumption, low folate intake, and high body mass index [72, 108]. High calcium intake, hormone replacement therapy, and use of nonsteroidal anti-inflammatory drugs are associated with a reduced risk. Perhaps the most interesting of risk factor associations involves cigarette smoking. The relationship between smoking and colorectal neoplasia has become known as the *smoking paradox* in that smoking is associated significantly with polyps, but its relationship with CRC is much weaker. A consistent observation has been the relationship between smoking and serrated polyps, which has been analyzed in a number of population-based studies [109]. Three independent studies have demonstrated a concordant pattern of higher risk estimates for serrated (hyperplastic) polyps than for conventional adenomas [110–112] in long-term and current smokers. When both serrated polyps and conventional adenomas were present [110–112], risks were higher still. The association of current smoking with serrated polyps begins very early in serrated neoplasia [113] and is the greatest in the distal colon [114], where malignant potential of the serrated polyps is low. The weak association between smoking and CRC has been explained by the relationship being dominated by the serrated pathway subset with *BRAF* mutation [115], which accounts for only 15% of CRC.

The role of smoking in serrated polyposis has not been extensively explored. In 2010, several reports added to the puzzle surrounding the smoking paradox. Initially it was demonstrated that current smoking is associated with a significantly higher polyp count in patients with serrated polyposis [11]. Also in 2010, a report suggesting a causative role for smoking was published on a small series of cases and showed a higher prevalence of current smokers amongst serrated polyposis patients than in the population [116]. Given that current smoking is associated with increased serrated polyps [114] and even serrated aberrant crypt foci in the general population [113], both the preceding observations in serrated polyposis patients are reasonable. 

Later in 2010, the authors of this current review reported that, even though current smoking was associated with increased polyp numbers, there was no significant effect on the risk of CRC in a case series of 151 patients with serrated polyposis [12], once again highlighting the smoking paradox. Given that the major association between smoking and CRC is largely confined to those CRCs with somatic *BRAF* mutation, and less than one-third of CRCs in serrated polyposis harbor a *BRAF* mutation, this is perhaps not unexpected, as *BRAF*-mutated CRCs constitute a minority of serrated polyposis CRCs. Of interest, however, an unexpected finding emerged regarding currently smoking females, who had a significantly decreased risk of CRC, after correcting for age and adenomas [12] (O.R 0.10, 95% CI 0.02 to 0.47, *P* = .004). Further, female patients who had ever smoked had an average age of onset for CRC of 63 years compared to those who had never smoked (50 yrs). Though the results did not reach statistical significance due to low numbers, a trend for delayed onset of CRC in female smokers was evident. In the population, female smokers with serrated pathway subset CRC are elderly onset. 

 The preceding observation is consistent with a biological mechanism similar to that reported in ulcerative colitis patients [117] and suggests that perhaps an inflammatory process may be responsible for neoplastic progression in serrated polyposis in a *subset* of female patients and that, similarly to ulcerative colitis, smoking may be anti-inflammatory. In the population, a study of risk factors for serrated polyps demonstrated that aspirin use decreased the risk of advanced proximal polyps, lending indirect support to this hypothesis [114]. Alternatively, confounding factors may be responsible for this finding, including unspecified sex-specific factors related to body mass index (BMI) or hormonal factors, as observed in the protective effects of smoking on endometrial cancer. This finding whilst perplexing cannot be ignored because it could potentially lead to a CRC-preventive modality for female patients with serrated polyposis *independent* of cigarette smoking and its attendant harms.

## 9. Serrated Polyposis as a Genetic Predisposition Syndrome

Serrated polyposis has many hallmarks of a genetic predisposition. These include an earlier age of onset of CRC, polyp, and cancer multiplicity, increased CRC risk in both patients and their relatives, and restricted ethnicity. An important clinical consequence associated with serrated polyposis is the increased risk of both CRC [26, 39, 43, 44, 54] and possibly extracolonic cancers [27, 118, 119] in the family setting of serrated polyposis patients. The risk to first-degree relatives of CRC has been estimated at fivefold greater than that of the general population [10]. WHO Criterion 2 (Text Box 2) addresses the evidence that serrated polyposis may occur in a familial context [25, 26, 40, 43, 48, 54, 61] and elevates the significance of smaller numbers of hyperplastic polyps in a first-degree relative of an individual with serrated polyposis. The genetic basis for serrated polyposis is yet to be determined, though small numbers of patients have reported mutations in *MUTYH *[90], *PTEN *[120], and *EPHB2* [121].

Biallelic *MUTYH* mutation is a phenotypically diverse disorder which appears to interact with the genetic background of the individual. In one-third of biallelic mutation carriers, there are no adenomas present [122]. In approximately 1% of patients with serrated polyposis, biallelic mutation of *MUTYH* can be demonstrated [123]. Conversely, when biallelic *MUTYH* mutation carriers are assessed, 18% meet the WHO criteria for serrated polyposis [90]. A recent report from Buchanan et al. [124] suggests that individuals with both *MUTYH*-associated polyposis (MAP) and serrated polyposis may be segregating two conditions with diverse modes of inheritance. The report describes a 56-year-old Caucasian male with >100 colonic polyps (approximately 50 conventional 10–15 mm adenomas predominating in the proximal colon and approximately 50 < 5 mm serrated polyps in the distal colon and rectum) who also demonstrated biallelic mutation for the two common European variants in *MUTYH *[124]. His mother had CRC of the sigmoid colon at 70 yrs. His 17-year-old symptomatic son who was not a biallelic mutation carrier had multiple 4 mm hyperplastic polyps in the rectosigmoid. The implications of this case report are that the risk to first-degree relatives of biallelic *MUTYH* mutation carriers (RR < 1.5) [125] is not as substantial as in serrated polyposis where the risk is fivefold greater than the population risk [10]. Therefore screening protocols in the setting described need also to consider the extra risk to first-degree relatives that serrated polyposis can pose. For this reason, detailed pathological examination of the polyps in patients with MAP is recommended to exclude coexisting serrated polyposis. Should serrated polyposis also be present, screening beyond siblings should be considered.

The evidence that serrated polyposis is a genetic predisposition is accumulating. Though multiple cases of serrated polyposis within a single family are rare [39, 54], the phenotype of multiple neoplasms, young-onset, and occasional affected sibships including consanguineous kindreds [26] suggest a pattern of inheritance consistent with an autosomal recessive or codominant mode [13, 126]. Codominant modes of inheritance result in an intermediate phenotype when one variant risk allele is present and a significantly altered phenotype in those where both alleles are variants.

## 10. Surveillance and Cancer Prevention Approaches

Several reports have suggested that malignant transformation in the serrated pathway may be unusually rapid in some clinical settings. Despite these observations, the apparent rapid evolution to cancer of advanced serrated polyps remains unproven and may be due to the difficulty of visualizing flat serrated lesions at colonoscopy. SSA/P progress very slowly to dysplasia in a population cohort [62]; however, in the syndromic patient this progression may be more rapid in an epigenetically abnormal environment. Hyman and colleagues reported 3 cases of serrated polyposis where CRC developed despite colonoscopy every 2 years [43]. Similarly, Azimuddin and colleagues reported that colonoscopy every 3 years was inadequate for some families with atypical serrated polyps [40]. Boparai et al. reported that 5 of 77 serrated polyposis patients developed a CRC whilst under surveillance for 5 years [9]. In four of five, the CRC arose within 12 months of a previous colonoscopy; however, many polyps had been left *in situ*. Currently, the issue of rapid evolution in serrated polyposis remains unresolved.

There are no clear guidelines though recommendations are evolving [127]. Frequent surveillance colonoscopy in the initial period after diagnosis both to allow endoscopic control of the polyps and to determine the nature and progress of the disease appears justified. Appropriate subsequent surveillance intervals can then be determined, but to avoid interval CRC this is unlikely to extend beyond 2-3 year intervals. Referral to a tertiary centre should be planned particularly if the polyp burden is difficult to control endoscopically and surgery is being considered.

## 11. Summary and Future Directions

Serrated polyposis is a condition with an increased CRC risk to both individuals and their relatives. An understanding of the mechanism of malignant transformation in serrated polyposis is still evolving, along with the risk factors which influence it [96]. Without a known germline sequence variant and estimated genetic penetrance, the identification and management of individuals and their families with a CRC predisposition syndrome become increasingly problematical. The prospect of a syndrome with a codominant mode of inheritance presents particular difficulties in that, although some individuals will present with a florid phenotype, such as that seen in serrated polyposis, first-degree relatives may have only a few polyps or none at all. The role of genetics departments, pathologists, and endoscopists in understanding the clinical picture for such families is likely to become increasingly important and interdependent. The challenge will be to determine if we can confidently identify and assign CRC risks to the different phenotypes of serrated polyposis, thereby allowing tailored clinical management with regard to the frequency of colonoscopic surveillance, the aggressiveness of polyp removal, and consideration of colonic resection.

##  Conflict of Interests 

The authors have no conflict of interests to declare with respect to this work.

## Figures and Tables

**Table 1:** Summary findings in serrated polyposis from publications reporting more than five cases (adapted from Buchanan et al., 2010) [11].

Author	Year	Cases (*n*)	Mean age at diagnosis (years)	Number of polyps observed	% with CRC	Family history of CRC
Kalady et al. [27]	2011	115	62	2-multiple	25%	38%
Buchanan et al. [11]	2010	126	49	5–150	40%	59%*
Boparai et al. [9]	2009	77	56	2–53	35%	NS
Carvajal-Carmona et al. [25]	2007	32	46	11-multiple	25%	59%
Chow et al. [26]	2006	38	44	20-multiple	26%	50%
Renaut et al. [61]	2001	28	58	20-multiple	29%	39%
Yeoman et al. [17]	2007	24	61	5-multiple	54%	17%
Ferrández et al. [28]	2004	15	53	15-multiple	0%	0%
Lage et al. [48]	2004	14	58	19–100	43%	33%
Hyman et al. [43]	2004	13	62	20-multiple	54%	38%
Rashid et al. [54]	2000	13	58	multiple	77%	38%
Leggett et al. [24]	2001	12	57	30–>100	58%	17%
Rubio et al. [23]	2006	10	61	6–159	70%	10%
Spjut and Estrada [56]	1977	9	53	Multiple	11%	NS
Williams et al. [29]	1980	7	37	50–150	0%	14%
Torlakovic and Snover [7]	1996	6	57	50–100	67%	NS
Place and Simmang [53]	1999	6	60	50–100	50%	14%

NS: not specified or unknown.

*Genetics clinic series.

**Table 2:** Prevalence of genetic and epigenetic alteration in the different subtypes of serrated polyps by combining all comparable data from [73, 75–84].

Polyp type	*BRAF *mutation	*KRAS *mutation	CIMP-H*	*MLH1 *methylation	*MGMT* methylation
HP					
Microvesicular	66.3%	12.3%	47.4%	39.5%	26.3%
Goblet-cell	20%	48.4%	14.3%	14.3%	0%
All subtypes	51.5%	22.1%	38.5%	27%	14.8%
SSA/P	83.9%	5.8%	75.9%	42.6%	25%
TSA	62.5%	22.4%	79.3%	47.5%	74.2%

*CIMP-H: high level of CIMP in polyps defined by ≥2/5 methylated markers from O'Brien et al. [76, 85].
